# Telemedicine in orthopaedics and trauma surgery during the first year of COVID pandemic: a systematic review

**DOI:** 10.1186/s12891-023-06194-3

**Published:** 2023-02-07

**Authors:** Ulf Krister Hofmann, Frank Hildebrand, Moritz Mederake, Filippo Migliorini

**Affiliations:** 1 Department of Orthopaedic, Trauma, and Reconstructive Surgery, RWTH University Hospital, Pauwelsstraße 30, Aachen, 52074 Germany; 2 Department of Trauma and Reconstructive Surgery, BG Klinik, University of Tübingen, Tübingen, 72076 Germany

**Keywords:** COVID-19, Pandemics, Coronavirus, Telemedicine, Orthopaedics, Trauma

## Abstract

**Purpose:**

Prior to the COVID-19 pandemic, telemedicine in orthopaedics and trauma surgery had mostly developed for joint arthroplasty, fracture management, and general pre- and postoperative care including teleradiology. With the corona-outbreak, telemedicine was applied on a broad scale to prevent assemblage and to guarantee access to medical care protecting critical areas. The purpose of the present study was to give an overview of the spectrum of clinical applications and the efficacy of telemedicine in orthopaedic and trauma surgery as published in times of the COVID-19 pandemic.

**Methods:**

All published studies investigating the application of telemedicine related to orthopaedics and trauma during the COVID-19 pandemic were accessed and screened for suitability. The primary outcome of interest was the efficacy of telemedicine in various clinical applications. The secondary outcome of interest was the spectrum of different applications in which telemedicine applications were investigated.

**Results:**

The literature search resulted in 1047 articles. After the removal of duplicates, 894 articles were screened of which 31 finally met the inclusion criteria. Dimensions that were described by studies in the literature to have positive effects were preoperative patient optimisation, the usefulness of telemedicine to correctly diagnose a condition, conservative treatment, willingness to and feasibility for telemedicine in patients and doctors, and postoperative/post-trauma care improvement. The efficacy of telemedicine applications or interventions thereby strongly varied and seemed to depend on the exact study design and the research question addressed.

**Conclusion:**

Various successful applications of telemedicine have already been reported in orthopaedics and trauma surgery, with a strong increase in scientific output during the COVID-19 years 2020–2021. Whether the advantages of such an approach will lead to a relevant implementation of telemedicine in everyday clinical practice should be monitored after the COVID-19 pandemic.

## Introduction

Telemedicine is defined as healthcare delivered from a remote location by means of telecommunications technology replacing face-to-face contact [1]. Telemedicine was mostly considered helpful to provide access to modern medicine in areas with great distances to cover to see the next specialist [2]. In the US, before an expansion of telehealth with the 1135 waiver on March 2020, Medicare refunded only patients in designated rural areas [3]. Telemedicine is safe and effective to deliver and it is used in many medical specialities [4]. Patients appreciate its convenience, shortened appointment delay, reduced travelling times, costs, and time off work [5]. In comparison to other medical disciplines, telemedicine demonstrated limited evolution and application in the field of orthopaedics and trauma surgery before the COVID-19 pandemic [6]. Telemedicine in orthopaedics and trauma surgery had mostly developed for joint arthroplasty, fracture management, and general pre- and postoperative care including teleradiology [7]. With the first corona wave on the rise, telemedicine was applied on a global scale to prevent assemblage and to guarantee access to medical care [8, 9]. With a general boost in online services during the pandemic, institutionalised services were re-organised, re-adapted, and made available for patients and medical staff, with easy to use interfaces and tutorials. Technologies broadly used during the COVID-19 pandemic were daily life domains, with artificial intelligence, video-based communication platforms, and remote computerised topographies affordable to everyone [10]. In this context, it can be hypothesised that also telemedicine developed and gained popularity. Hence, several clinical investigations on telemedicine have emerged in the past years [11–15].

The purpose of the present systematic review was to investigate the development of telemedicine during the first year of the COVID-19 pandemic in orthopaedic and trauma surgery, exploring its clinical application and efficacy. It was hypothesised that the use of telemedicine during the first year of the COVID-19 pandemic gained popularity and efficacy, providing an alternative to face-to-face consultation and reducing within hospital patient turnover and assembly.

## Material and methods

### Search strategy

This systematic review was conducted according to the Preferred Reporting Items for Systematic Reviews and Meta-Analyses: the 2020 PRISMA statement [16]. The following algorithm was preliminarily established:

- Population: orthopaedic and trauma patients;
- Intervention: telemedicine application;
- Outcomes: development, clinical application, and efficacy;
- Timing: during COVID pandemic.

In February 2022, PubMed, Web of Science, Google Scholar, and Embase were accessed restraining the search to articles published from January 1, 2020, until December 31, 2021. The following keywords were used in combination using the Boolean operator AND/OR: *telemedicine(ALL FIELDS)* AND *orthopaedics(ALL FIELDS)* OR *trauma(ALL FIELDS)* OR *traumatology(ALL FIELDS).* No additional filters were set for the literature search. Two authors (UKH; MM) independently performed the database search. All the resulting titles were screened per hand and, if suitable, the abstract was accessed. The full text of the abstracts which matched the topic was accessed. A cross reference of the bibliography of the full-text articles was also screened for inclusion. Disagreements were debated and settled by consensus.

### Eligibility criteria

All published studies investigating the application of telemedicine during the COVID pandemic were accessed. Only articles available in English were eligible. Original studies with a level of evidence of I to IV according to the Oxford Centre of Evidence-Based Medicine [17] were considered. Reviews, opinions, letters, and editorials were not considered. Animal studies, in vitro-, biomechanical, computational, and cadaveric studies were not eligible. Only studies published during the period 2020–2021 were included. Only studies related to orthopaedics and trauma surgery were eligible.

### Data collection and outcome of interest

The objective of the present systematic review was to provide an overview of the development of telemedicine during the COVID-19 pandemic in orthopaedic and trauma surgery, exploring its clinical application and efficacy. The following data were extracted and retrieved in a Microsoft Office Excel Version 16.69 (Microsoft Corporation, Redmond, US): name of the first author, year and journal of publication, level of evidence, study design, country of the study, purposes and main findings.

## Results

### Study selection

**Fig. 1:**
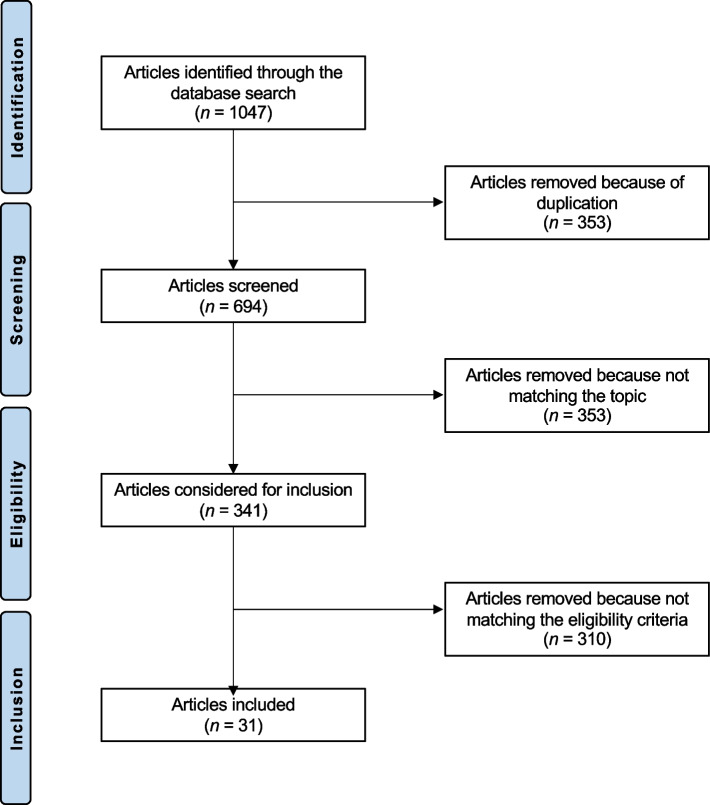
Flow chart of the literature search

The literature search resulted in 1047 articles. After the removal of duplicates, 694 articles were screened. Of them, 353 studies did not focus on telemedicine applications in the field of orthopaedic and trauma surgery during the COVID-19 pandemic. A further 310 studies were excluded for the following reasons: language limitation (*n* = 33), not clinical studies (*n* = 171), and not being published during the period 2020–2021 (*n* = 106). Finally, 31 publications met the inclusion criteria and were included in the present systematic review (Fig. 1).

### Study characteristics and results of individual studies

Key interests in the published works were preoperative patient optimisation, the efficacy of telemedicine in diagnosis, follow-up of conservative and surgical treatments, willingness to and feasibility for telemedicine in patients and medical staff, and postoperative/post-trauma care improvement. Two studies found a positive impact of telemedicine in preoperative patient optimisation [18, 19]. Four studies evaluated the willingness to and feasibility of performing telemedicine in patients and doctors [20–23], and one study investigated its application in diagnoses [24]. Two studies evaluated the implementation of telemedicine for conservative treatments, including rotator cuff ailments [25, 26]. For chronic musculoskeletal spinal conditions, results were unambiguous and warrant further investigations [25]. Most studies in the field of orthopaedics investigated the efficacy of telemedicine in postoperative care [27–32]. In the field of trauma, the success of prevention programs was evaluated in two studies [33, 34], pre-treatment optimisation in one study [35], and conservative treatment/outpatient care in four studies [36–39]. One further study described the necessity to use high quality photographs to correctly formulate diagnoses via telemedicine [40]. The majority of studies deal with postoperative or post-trauma care improvements [41–47]. Generalities, purposes, and main findings of the studies which investigated the application of telemedicine during the COVID-19 pandemic in orthopaedics and trauma surgery are reported in Tables 1 and 2, respectively.

**Table 1:** Clinical studies from the field of orthopaedics reporting on telemedicine from 2020 and 2021

Author, year	Journal	LoE	Design	Country	Purpose of the study	Main findings
An et al., 2021 [18]	*Int J Environ Res Public Health*	II	RCT	Korea	Analyses the effects of preoperative telerehabilitation muscle strength, range of motion, and functional outcomes (WOMAC-score, Timed Up and Go Test) in patients intended for total knee arthroplasty. After a preoperative 3 week intensive exercise program, significantly better results were obtained in the intervention group 4 weeks postoperatively when compared to controls who did not receive such preoperative training	Successful preoperative telerehabilitation program before total knee arthroplasty
Pabinger et al., 2021 [24]	*Int J Med Inform*	II	RCT	Austria	Evaluates the usefulness of a mobile healthcare communication app with respect to diagnosis and treatment in outpatient care of general surgery. The parameters used for the app were uploaded by doctors seeing the patient on-site. With this setup the diagnosis and treatment recommended by the telemedicine doctor showed high congruency with those formulated by the on-site doctor	Successful tele-diagnosis and -treatment in outpatient care of general surgery
Mehta et al., 2020 [31]	*JAMA Netw Open*	I	RCT	USA	Looks into the effects of activity monitoring and bidirectional text messaging on the rate of discharge to home and clinical outcomes in patients obtaining knee or hip replacement. Only rehospitalisation rate reduced in the intervention group, while discharge status and average daily step count were comparable among both groups	Activity monitoring and bidirectional text messaging
Malliaras et al., 2020 [26]	*JMIR Mhealth Uhealth*	III	RCT	Australia	Assesses the feasibility of a 12-week internet-delivered intervention for rotator cuff-related shoulder pain. The authors compared the treatment regimes of advice only, internet-delivered evidence-based exercise and education, and this internet-delivered care with group-based telerehabilitation including a weekly group teleconference session	Successful adherence of an internet-delivered intervention for rotator cuff-related shoulder pain
Seward et al., 2020 [19]	*J Orthop Surg Res*	II	RCT	USA	Describes a study protocol of an inaugurated study aiming to evaluate weight loss before total joint arthroplasty using a remote dietician and mobile app	Study protocol
Bell et al., 2020 [27]	*Sensors*	II	RCT	USA	Evaluates the feasibility of a wearable remote rehabilitation monitoring platform (interACTION) for the remote management of rehabilitation after total knee arthroplasty	Successful feasibility study for a tele-rehabilitation platform after total knee arthroplasty
Claassen et al., 2020 [20]	*BMC Med Inform Decis Mak*	I	RCT	Netherlands	Looks into the effects on patients’ satisfaction of a stand-alone mobile and web-based educational intervention compared to usual preparation of a first orthopaedic consultation in patients with osteoarthritis of the knee or hip. While the digital applications did not lead to a higher patient satisfaction with the consultation, they influenced knowledge on osteoarthritis	Higher patient knowledge on osteoarthritis after a web-based educational intervention
Higgins et al., 2020 [28]	*Arthroscopy*	II	RCT	Canada	Evaluates if a mobile app can reduce the need for in-person visits after anterior cruciate ligament reconstruction. In the mobile app group patients frequented less often the physician while achieving the same satisfaction, complication rates and clinical outcomes	Successful telemedicine application after anterior cruciate ligament reconstruction
Kane et al., 2020 [30]	*J Shoulder Elbow Surg*	II	RCT	USA	Investigates safety, efficacy and socioeconomic benefits of telehealth as a platform for postoperative follow-up after arthroscopic rotator cuff repair in comparison to a control group receiving regular postoperative care. While pain scores and satisfaction were similar in both groups, patients in the telehealth group expressed a stronger preference for telehealth	Postoperative telehealth after rotator cuff repair leading to high patient acceptance
Huang et al., 2020 [29]	*Sensors*	III	controlled clinical trial	Taiwan	Proposes a sensor-based system to effectively remotely monitor rehabilitation progress after total knee arthroplasty	Testing of a remote monitor system for rehabilitation after total knee arthroplasty
Pronk et al., 2020 [32]	*JMIR Mhealth Uhealth*	II	RCT	Netherlands	Analyses the effects of the PainCoach app on postoperative pain control and opiate use in patients who received total knee arthroplasty. With comparable pain scores as in a control group, the opiate consumption was reduced in the intervention group	Successful application of an app to reduce postoperative opioid consumption
Cottrell et al., 2021 [25]	*J Telemed Telecare*	III	prospective non-randomised clinical trial	Australia	Compares telerehabilitation with in-person care for patients with chronic musculoskeletal spinal conditions. The unambiguous results of the study warrant further investigation	Telerehabilitation for chronic musculoskeletal spinal conditions. No unambiguous results
Scherer et al., 2021 [22]	*Injury*	IV	Survey	Switzerland, Germany	Assesses questionnaire-based the willingness of orthopaedic patients to perform video consultations. Older patients are less eager to use remote consultation. The most frequently stated disadvantage was the lack of physical examination	Younger patients are more prone to use video consultations
Omari et al., 2021 [21]	*Telemed J E Health*	IV	Survey	USA	Survey with high satisfaction rates with telemedicine. Patients are more confident in follow-up visits and when also using the video channel	Survey reporting high satisfaction rates with telemedicine
Versluijs et al., 2021 [23]	*Telemed J E Health*	II	RCT	USA	Randomised controlled trial investigating the effect of previsit phone calls from the surgeon. There is no effect regarding decision conflict or perceived empathy. However, the surgeons felt that 91% of the in-person visit can be replaced by phone calls	Previsit phone-calls did not reduce decision conflict

**Table 2:** Clinical studies from the field of trauma surgery reporting on telemedicine from 2020 and 2021

Author, year	Journal	LoE	Design	Country	Purpose of the study	Main findings
Ortiz-Piña et al., 2021 [47]	*Int J Environ Res Public Health*	II	Prospective non randomised clinical trial	Spain	Analyses the effects of a multidisciplinary 12 week postoperative tele-rehabilitation program on functional recovery in elderly patients with hip fractures when compared with a home-based in-person rehabilitation considered standard of care in Spain. Higher functional independence measure scores and better Timed Up and Go Test results were found in the intervention group	Tele-rehabilitation in patients with hip fractures showing higher functional independence than in standard of care group
Delbaere et al., 2021 [33]	*BMJ*	I	RCT	Australia	Tests if a specific exercise program intended for older people and digitally delivered through an app (StandingTall) can reduce the rate of falls. No difference was seen in the first 12 months, but a slightly reduced fall rate over 24 months when compared with the control group	App based exercise program showing no reduced rate of falls in elderly people after 12 months
Binder et al., 2021 [42]	*Contemp Clin Trials*	II	clinical trial	USA	Study protocol with the intention to evaluate the six minute walk distance and other parameters after testosterone gel application and a digital home exercise program after hip fracture in the elderly	Study protocol
McCarty et al., 2021 [46]	*JAMA Netw Open*	I	RCT	USA	Examines if a collaborative care treatment is associated with improvements in patients with post concussive symptoms. The interventions provided were largely based on telehealth applications and succeeded in better improving symptoms in patients over one year	Telehealth applications in patients with post concussive symptoms successfully improved symptoms over one year
Wohlmann et al., 2021 [48]	*Telemed J E Health*	II	RCT	Germany	Describes positive effects of a medical emergency data set on the comprehensiveness of a physician’s documentation and handover process to the emergency department of a hospital	Positive impact of a digital medical emergency data set
Ariza-Vega et al., 2021 [41]	*Phys Ther*	II	clinical trial	Spain	Evaluates the family caregivers’ perspectives of the recovery process of the elderly patients in their care with hip fracture comparing an online rehabilitation program with the standard of care in Spain	Main reasons for caregivers and their family member to chose a telerehab program were to enhance recovery after fracture, gain knowledge for managing at home, and because of the convenience of completing the exercises at home
Gilmore et al., 2020 [45]	*Womens Health Issues*	II	RCT	USA	Compares the effectiveness of prolonged exposure therapy in posttraumatic stress disorder after military sexual trauma in women with a special focus on this therapy delivered either in person or via telemedicine	Dropout rate in prolonged exposure therapy in posttraumatic stress disorder was independent of whether it was delivered in person or via telehealth
Campbell et al., 2020 [35]	*J Trauma Stress*	III	multicentre study	USA	Describes that pretreatment social support moderates the association between cognitive processing therapy duration and changes in posttraumatic stress disorder in a telemedicine-based collaborative care intervention for rural veterans	The connection between CPT treatment duration and treatment outcomes may be stronger for veterans with higher levels of pretreatment social support
Jeong et al., 2020 [40]	*JMIR Mhealth Uhealth*	II	RCT	China	Describes the necessity to use high quality photographs for correct remote diagnosis via telemedicine of dental trauma and that already simple instructions suffice to improve image quality	Simple instructions and good quality photos largely improve quality image interpretation in telemedicine
Rietdijk et al., 2020 [37]	*J Speech Lang Hear Res*	II	RCT	Australia	Investigates the effectiveness of social communications skills training (TBIconneCT) for people with traumatic brain injury and their communication partners when delivered either in-person or via telehealth. Similar improvements in communication skills were found in both groups	Telehealth delivery of social communication skills training can be equally effective as in person training
Pas et al., 2020 [34]	*Br J Sports Med*	I	RCT	Netherlands	Evaluates the effectiveness of an e-health prevention program on reducing tennis injury prevalence. Over a four months period, no difference was observed between the control group and the group using the TennisReady program	No effect of an e-health prevention program on reducing tennis injury prevalence
Rietdijk et al., 2020 [38]	*J Head Trauma Rehabil*	II	RCT	Australia	Evaluates the efficacy of social communications skills training (TBIconneCT) for people with traumatic brain injury when delivered either in-person or via telehealth. Outcomes reported by the survivor and close communication partner showed no relevant difference between both delivery channels	Telehealth delivery of social communication skills training can be equally effective as in person training
Coronado et al., 2020 [44]	*Phys Ther Sport*	III	RCT	USA	Assesses the feasibility (*n* = 8) of a telephone-based cognitive-behavioural-based physical therapy intervention for improving postoperative recovery after anterior cruciate ligament reconstruction	Satisfied patients after anterior cruciate ligament reconstruction receiving a telephone-based cognitive-behavioural-based physical therapy intervention
Téot et al., 2020 [39]	*Int J Low Extrem Wounds*	II	RCT	France	Compares the outcomes of patients with complex wounds who received home wound care from a local clinician instructed by a remote wound care expert via telemedicine versus the results from patients who received their wound care directly from the specialist. No significant difference was found between both groups	Successful wound management via telemedicine by means of a local clinician instructed remotely by a wound care expert
Buccellato et al., 2020 [43]	*Mil Med*	II	RCT	USA	Feasibility trial of a virtual rehabilitation program (BBVR) in patients with acquired brain injuries	All of the participants and providers reported moderate to high levels of utility, ease of use and satisfaction with the BBVR system
Hart et al., 2020 [36]	*Neuropsychol Rehabil*	II	RCT	USA	Compares treatment for depression or anxiety in traumatic brain injury patients when delivered by two different protocols, both of which included daily motivating text messages (SMS)	Only moderate effects were obtained by the SMS

## Discussion

Various applications of telemedicine have been reported in orthopaedics and trauma surgery, with a strong increase in scientific output during the years 2020–2021. Whether the advantages of such an approach will lead to a relevant implementation of telemedicine in everyday clinical practice should be monitored after the COVID-19 pandemic. A broad range of different clinical applications of telemedicine was investigated during the first year of the pandemic.

The efficacy of telemedicine applications or interventions thereby strongly varied and seemed to depend on the exact study design and the research question addressed. Classen et al. [20] for example compared the effect of an educational teleintervention (eHealth tool) to the usual elective consultation in patients with osteoarthritis of the knee or hip in a randomised controlled trial involving 286 patients. The authors concluded that an educational teleintervention does not improve patient satisfaction with the consultation, but it increases cognition about osteoarthritis [20].

Functional recovery was investigated by Ortiz-Piña et al. [47], who evaluated the influence of a multidisciplinary telerehabilitation program on functional recovery in elderly patients following surgical management for hip fracture in a single-blinded, non-randomised clinical trial. Twelve weeks of telerehabilitation program (35 patients) were compared to a control group receiving usual care (35 patients) [47]. Participants who used the telerehabilitation program had higher Functional Independence Measure scores and better performance in the Timed Up and Go Test compared with the control group, but no difference in the Short Physical Performance Battery [47]. Also in pain management, positive data were published: Pronk et al. [32] performed a randomised clinical trial to evaluate the introduction of a mobile application to manage pain in 71 patients following total knee arthroplasty during the first two weeks postoperatively. The authors stated that this method is effective to manage pain and that it reduces opioid consumption [32]. Teot et al. [39] conducted a randomised controlled trial comparing the efficacy of complex wound management. In their study, the authors evaluated complex wound management performed by untrained staff guided by an off-site wound care expert via telemedicine (89 wounds) versus professional staff at home (59 wounds) versus professional staff in the clinic (35 wounds) [39]. The authors found no difference between the three modalities, stating that telemedicine may represent a reliable alternative [39]. Similar results could be obtained by Westley et al. [49] when comparing the accuracy of virtual versus face-to-face assessment in hand trauma: there were no significant differences [49]. Another important issue is patient satisfaction, as it is crucial for compliance. Several studies are available all of them showing high patient and physician satisfaction, however not equal to face-to-face examination [21, 50–52]. Especially older patients show lower acceptance rates [22, 53]. Looking into a third-party perspective, Ariza-Vega et al. [41] investigated 44 family caregivers who had an older family member with a previous hip fracture. The authors stated that telemedicine should also address the family caregivers, who represent an essential component of recovery after hip fracture by providing emotional and physical support [41].

While previously being used only in a limited way in the context of mostly pre- and postoperative care, the use of telemedicine appears to have strongly increased during the COVID-19 pandemic. This is also reflected in the number of articles in PubMed resulting from “telemedicine AND orthopaedics”. Indeed, in 2018 and 2019 only 62 and 65 articles were retrieved, this number increased to 233 and 273 articles in 2020 and 2021, respectively. The same applies to the search terms “telemedicine AND trauma”: from 141 and 128 articles in 2018 and 2019 to 322 and 270 in 2020 and 2021, respectively. The evident problem of telemedicine in orthopaedics and trauma is the high relevance of manual clinical examination. Nevertheless, several applications are successful, such as postoperative telerehabilitation after total knee arthroplasty [54], or post-operative telemedicine encounters following rotator cuff repair [30]. Especially follow-up appointments are favourable for telemedicine since the patient-physician-connection is already stable and laboratory or surgery results can be discussed virtually as well [21, 51, 55]. In addition, single parameters such as the range of motion can be reliably evaluated by telemedicine in various joints [56–58]. First studies evaluating patient and surgeon satisfaction after online consultations showed promising results, with patient satisfaction exceedingly clearly that of surgeons [21, 50–52, 59]. Of note, satisfaction is a subjective outcome parameter and does not reflect the actual quality provided by telemedicine. With still further improving digital infrastructure the role of telemedicine is likely to increase in the coming years, especially if conditions like the COVID-19 pandemic turn up again. In addition, the still increasing specialisation in the field might lead to higher use of online services including virtual reality. To define, however, conditions and circumstances where to adequately apply telemedicine is still a challenge to face.

The present study has several limitations. While the present study highlights the findings of those studies published in the context of telemedicine during the COVID-19 pandemic, it does not selectively display information on changes directly measured in the context of the pandemic itself. It thus only provides a current picture of the general use of telemedicine, its possibilities and weaknesses. It neither analyses its suitability per-se to replace direct patient-doctor contact nor does it describe actual changes in use as a result of the pandemic. These articles are likely to emerge in the upcoming months and shall then be analysed accordingly.

## Conclusion

The use of telemedicine increased during the COVID-19 years 2020–2021 in orthopaedics and trauma surgery. Key interests in the published works were preoperative patient optimisation, the efficacy of telemedicine in diagnosis, follow-up of conservative and surgical treatments, willingness to and feasibility for telemedicine in patients and doctors, and postoperative/post-trauma care improvement. Whether the advantages of telemedicine will lead to its relevant implementation in everyday clinical practice should be monitored after the COVID-19 pandemic. New digital possibilities, including artificial intelligence and virtual reality, should also be implemented and carefully evaluated.

## Data Availability

The datasets generated during and/or analysed during the current study are available throughout the manuscript.

## Abbreviation

PRISMA: Preferred Reporting Items for Systematic Reviews and Meta-Analyses
